# An Exploration of the Biochemistry of Mustard Seed Meals: A Phytochemical and In Silico Perspective

**DOI:** 10.3390/foods13244130

**Published:** 2024-12-20

**Authors:** Shivanshu Garg, Kanchan Gairola, Himanshu Punetha, Saurabh Gangola

**Affiliations:** 1 Department of Biochemistry, College of Basic Sciences and Humanities, GBPUAT, Pantnagar 263145, Uttarakhand, India; kgairola0122@gmail.com (K.G.); hpunetha15@gmail.com (H.P.); 2 Department of Microbiology, Graphic Era University, Dehradun 248001, Uttarakhand, India

**Keywords:** coat color, functional properties, molecular docking, mustard seed meal, mustard oils, pepsin digestibility

## Abstract

The present investigation deals with comparisons drawn among three types of different mustard seed coat colors, namely, Black (*Brassica nigra*), Brown (*Brassica juncea*), and White (*Sinapis alba*), with respect to protein’s bio-availability through pepsin digestibility, with and without the involvement of major anti-nutritional factors (glucosinolate type AITC, Allylisothiothiocyanate) and relative food functions. These are validated by means of crude protein determination, precipitated protein isolate preparation for evaluating the fat absorption capacity (FAC), emulsifying activity (EA), emulsion stability (ES), whippability, foam stability (FS), the nitrogen solubility index (NSI), and the protein dispersibility index (PDI). The results indicate that the partial removal of glucosinolates from brown mustard (0.91 to 0.31%), black mustard (0.74 to 0.31%), and white mustard (0.58 to 0.30%) improved protein’s digestibility, as analyzed through a pepsin assay, with values of 12.84, 12.60, and 4.53% in brown, black, and white mustard, respectively. Among functional properties, the highest FAC, whippability, foam stability, and NSI values were noted in the brown mustard seed meal, derived from precipitated protein isolates, while EA and PDI were the highest in white mustard seeds, and black seeds possessed the highest ES value. Interestingly, these mustard seed meals are limited in their consumption, albeit by virtue of the different phytochemical and food functional parameters that are being elucidated here. The present research shows the relevance of different food functional properties and the presence of anti-nutritional factors, and uses protein-digestibility tests, which are important deciding parameters for enhanced food consumption in animal diets. Additionally, targeted molecular and protein–protein docking results revealed how and why the mustard seed meals are limited in their consumption by virtue of various metabolite interactions. This thereby opens the gateways to many required in vivo and in silico future research insights among AITC–pepsin, AITC–myrosinase, pepsin–myrosinase, and cruciferin–myrosinase complexes. Consequently, the metabolic pathways governing AITC involved in the glucosinolate–myrosinase system need to be studied in depth for a better understanding of in vivo AITC metabolism. This knowledge can guide future studies in improving the health benefits of mustard seeds and seed meals while addressing their consumption limitations.

## 1. Introduction

Mustard holds an important place among the spices, as it contains a wide array of phytochemicals and, moreover, is one of the oldest oilseed crops. Widely grown all over the world, cultivation records show its having been present on Earth since 3000 B.C. [1]. Previously known as Capparales, Brassicales is the order followed by the family Brassicaceae, previously known as Cruciferae, to which the mustard plants belong under the taxonomical hierarchy [2]. Key botanical characteristics of this plant are its four sepals (at the middle position of its flowers) and petals (arranged in a crossform pattern). The diverse array of organosulfur compounds are key biochemicals belonging to this family, especially glucosinolates.

The anti-nutritional factors present in mustard are phytic acid, glucosinolates, and sinapine, and these are the glucosinolates whose breakdown products are responsible for the pungency of mustard oils [3]. The glucosinolates present in seeds or oils are broken down by the enzyme myrosinase (EC: 3.2.1.147) into several breakdown products (isothiocyanates, nitriles, thiocyanates, and oxazolidine-2-thiones), of which AITC is a major one [4]. AITC inhibits digestive enzymes like pepsin, which leads to an overall negative impact on protein digestion. This hinders amino acid and mineral bio-availability [5,6]. The presence of these anti-nutritional factors limits the consumption of mustard seeds as food, irrespective of the high protein content which, if otherwise consumed in high amounts, leads to allergenic reactions [7]. The necessity of sulfur incorporation from soils to the formation of glucosinolates (sulfur-containing compounds) in Brassicaceae plants lies in the biochemical pathways. It has been experimentally noticed that sulfur-enriched soils, as compared to sulfur-deficient soils, lead to a higher total glucosinolate content in mustard plants, as compared to plants grown under a deficit regarding sulfur content in soils. The status of the sulfur content should be kept in a narrow range so as not to attain metabolic fluctuations in the total glucosinolate content of the plants [8]. Mustard, as a spice, contains varied amounts of biochemical metabolites like sinigrin, sinalbin, quercitin, kaempferol, and fatty acids, which influence its role as a food ingredient (spice) all over the world. Aside from the nutritional perspective, the property which influences the use of an ingredient in food is defined as its functionality. Defining the functional properties, it is observed that the protein content governs the physicochemical characteristics of food and the changes occurring during preparation, processing, and storage [9]. Studying the functional properties requires precipitated protein isolates, which are crude preparations of protein molecules clustered together. The structure, conformation, and composition of the protein ingredients are reflected by the functional properties. In seeds of the Brassicaceae family, the cruciferin is a storage protein that exhibits antioxidant, antimicrobial, and emulsifying properties, providing nutritional benefits, potential health advantages against cancer and cardiovascular diseases, and applications in the food industry, with its unique structure comprising α and β subunits rich in essential amino acids like methionine and cysteine [10]. Along with cruciferin, the napin protein is also distributed in seeds of the plants belonging to this family [11]. The functional properties are dependent on ionic strength, and temperature, and undergoing a type of processing like isolation or dehydration [12]. Functional properties reflect the composition and conformation of the protein source and processing treatment, and can be used to describe their behavior in food systems. Typical functional properties include FAC, EA, ES, whippability, FS, NSI, and PDI [13].

The amount of oil retained by a protein mixture after a thorough mixing with oils and centrifugation is classified as FAC. Fat absorption can be explained by the oil’s physical entrapment through capillary action with hydrophobic groups interacting on the surface of the protein [14]. EA and ESI are conducted to estimate the emulsifying properties of proteins. EA is expressed mathematically as the percentage volume of the emulsified layer of the emulsion prior to centrifugation [15,16]. The emulsion’s ability to stand with heating until its breakdown is represented in terms of the ESI [17]. The calculation of ESI corresponds to the remainder of the EA expressed as a percentage after heating [18]. The foaming ability, or whippability, and foam stability represent the foaming properties. The size of the increase in protein dispersions due to the gas introduction is measured as the whippability, while the ability to retain the formed foam to its maximum volume over time is described as foam stability [19]. To decipher quickly the functional properties of proteins, the PDI and NSI are suitable measures of experimentation [20,21]. Both these parameters are differentiated by stirring speeds. NSI is expressed as the percentage of water-soluble nitrogen to total nitrogen under slow stirring. PDI is expressed as the amount of dissolved protein as the percentage of total protein under fast stirring [22,23]. The components of the biological system aiding in digestion includes gastric juice produced by the stomach and comprises mucus, hydrochloric acid, and pepsin [24]. Among these, pepsin is the enzyme whose work is to breakdown large proteins into smaller amino acids and peptide so as to make the absorption process easy for the small intestine [25]. The higher content of glucosinolates leads to pepsin inhibition [6] so the increase and decrease in pepsin activity is correlated here with the number of glucosinolates present in mustard seed types by their subsequent analysis.

Mustard oils contain a wide array of saturated and unsaturated fatty acids and thus serve the role of a cooking ingredient [26]. FTIR spectroscopy is used to identify the presence of bonds such as the symmetric and asymmetric carbon–hydrogen single bond, ester bonds of carbonyl groups, and single carbon–oxygen bonds by analyzing the infrared spectrum of emission or absorption [27], in the three diverse mustard types described here.

The biochemical and functional analysis conducted here highlights the significance of various mustard metabolites, which enable its consumption despite the presence of anti-nutritional factors, albeit to a limited extent. The mustard seeds and seed meals have a high protein content but cannot be utilized due to the presence of anti-nutritional factors. Among the anti-nutritional factors, glucosinolates essentially limit the consumption and primary glucosinolate breakdown product (GBP); the AITC produced after the enzymatic action of myrosinase is taken here into consideration, showing its potential involvement with the protein digestibility analysis mediated by pepsin. The work undertakes only seed meals obtained after defatting because they are primarily used as feed for poultry, cattle, and fish. AITC, as the major GBP, is estimated in different mustard seed meal types. Additionally, an analysis of the in vitro protein digestibility is performed on precipitated protein isolates obtained from seed meals with a reduced GBP level and those with naturally retained GBP levels. The protein–protein docking of pepsin–myrosinase and cruciferin–myrosinase shows the relevance of the protein-digesting enzyme pepsin, found in the biological systems of vertebrates, to the major mustard protein cruciferin. This interaction is analyzed in relation to the GBP-producing enzyme myrosinase, present in mustard seed. The targeted molecular docking of AITC with pepsin reveals a weak molecular interaction, suggesting that AITC may be consumed but only in limited amounts. Similarly, the docking of AITC with myrosinase indicates weak binding, implying the possibility of regulatory metabolic controls in the functioning of myrosinase. This suggest that AITC, a byproduct catalyzed by one of myrosinase’s substrates, may influence the enzyme’s activity at a regulatory level. The present investigation aims to explore the limited-scale use of mustard seed meals in the vertebrate diet through phytochemical experiments, functional properties, and in silico analysis. To reflect the biochemistry aspects, soil sulfur estimation was also conducted as glucosinolate biosynthesis depends on the concentration in the soil. The sulfur level in the soil was consistent across all three mustard seed types sown in the field, ensuring minimal variation. The Fourier-transform infra red (FTIR) spectroscopy analysis was performed on mustard-oil-extracted seeds, as black, brown, and white mustard oils are commonly used for culinary purpose. The seeds were harvested after sowing and, subsequently, used for experimentation. This experimentation analysis aims to pave the way for further research into the biochemistry of anti-nutritional factor interactions with nutritional metabolites present in mustard seed types. This study is the first of its kind, providing a comparative analysis of three distinct mustard types widely utilized globally.

## 2. Materials and Methods

In the present investigation, mustard seeds were defatted using soxhlet assembly [28] and these defatted seed meals were then subjected to determination and lowering of GBP. For protein digestibility analysis by pepsin and functional property estimation, precipitated protein isolates were prepared from defatted seed meals. Additionally, the sulfur content of soils where the mustard seeds were sown was analyzed. FTIR analysis was also conducted on mustard oils extracted after defatting the mustard seeds.

### 2.1. Collection of Seed Material

The seeds of black, brown, and white mustard required for experimentation were provided by Director Research, Experiment Station, Norman E. Borlaug Crop Research Centre, GBPUAT, Pantnagar, India; 100 g of seeds from each mustard type were harvested in March 2023, following sowing in late September.

The execution of experiments was carried out at Lab 105 A, Metabolite Research Laboratory, Department of Biochemistry, College of Basic Sciences and Humanities, Govind Ballabh Pant University of Agriculture and Technology, Pantnagar, India. The required chemicals including AITC (Product No. 36682) were ordered from Sigma-Aldrich (Burlington, MA, USA) and concerned instrumentation facilities available were utilized.

### 2.2. Experimentation

#### 2.2.1. Phytochemical Estimation

(a) Determination of Crude Protein and Sinapine Content

For crude protein estimation, micro-Kjeldahl method was employed to calculate % nitrogen and factor 6.25 was employed to find out crude protein (%) [3]. The sinapine content was calculated using reflux assembly [29]. Absorbance was read at 330 nm for sinapine estimation, using methanol as a blank. The sinapine percentage was calculated with the following formula:

% sinapine = (2.184 × Absorbance × 10)/(sample weight in grams)

(b) Determination and Lowering of Glucosinolate Breakdown Products

Titrimetric methods were used to analyze the concentration of GBP from different mustard seed meals, and the subsequent reduction was evaluated [5,30]. The modification includes reducing the incubation time from 8 h to 3 h at 80 °C, followed by an additional incubation period at 4 °C after moderate air drying for 2 h.

The amount of AITC was measured using a titrimetric method. For this, 5 g of raw mustard cake was added to 12.5 mL of absolute ethanol and 237.5 mL of distilled water in a 500 mL distillation flask. The mixture was subjected to steam distillation, and 150 mL of the distillate was collected in a solution containing 25 mL of 0.1 N silver nitrate and 10 mL of 10% ammonium hydroxide. This distillate mixture was then boiled for 1 h in a water bath under air reflux, allowed to cool, and brought up to a final volume of 250 mL before filtering. From the filtrate, 100 mL was titrated with standard ammonium thiocyanate solution under acidic conditions, with a few drops of ferric ammonium sulfate used as an indicator. A blank titration was also performed to calculate the concentration of AITC.

(c) Protein Extraction and Preparation of Protein Isolates

Protein extraction was conducted following the method described by Marnoch and Diosady, with slight modifications [31]. To assess the protein extractability, 20 g of defatted mustard cake powder were mixed with an aqueous NaOH solution, using a solvent-to-cake ratio of 18:1. The solution’s pH was adjusted with a phosphate buffer, at 8.5. After extraction, the mixture was centrifuged at 12,000× *g* rpm to separate the liquid extract from the solids. The liquid fraction was collected by decanting and vacuum-filtered through Whatman 41 paper. The residual solids were washed twice with distilled water, and the wash solutions were combined in the same flask. Extractability was calculated as the ratio of the protein mass in the extract to that in the initial 20 g sample.

Protein was precipitated from the extract by adding a 1 M HCl solution, adjusting the pH to 5, and allowing the mixture to sit overnight at 5 °C. The precipitated protein isolate (PPI) was then collected by centrifugation at 10,000 rpm for 15 min using a Kokusan H2000 series centrifuge (Karnataka, India). The PPI was subsequently washed with water, freeze-dried, and stored at 5 °C for future analysis.

(d) Protein Digestion Analysis of Precipitated Protein Isolates by Pepsin

Protein extraction was carried out from mustard cakes, and precipitated protein isolates were recovered [31,32]. Further, in vitro protein digestibility of mustard cakes with lowered and unchanged concentration of glucosinolates was carried out [33,34]. The in vitro protein digestibility of PPI recovered from lowered and non-lowered GBP cake was assessed. A 2 g sample was combined with 490 mL of distilled water and 1.5 g of pepsin. Following this, 10 mL of 25% HCl was added, and the solution was incubated at 37 °C for 24 h. After incubation, the residue was collected and the non-digestible nitrogen content was determined using the micro-Kjeldahl method. The sample was then subjected to 8 h incubation at 37 °C with another 10 mL of 25% HCl. The reaction was halted by adding 15 mL of 10% trichloroacetic acid. Finally, the mixture was filtered and rinsed with distilled water.

(e) FTIR Analysis of Mustard Oils

FTIR analysis of mustard oils was carried out through Thermo Scientific (Waltham, MA, USA) Smart Multi-Bounce HATR system in Department of Biophysics, CBSH-GBPUA&T, Pantnagar. The sample was prepared by placing a small quantity of mustard oils (2 mL) on the FTIR ZnSe crystal, ensuring the surface was fully covered without air bubbles. For transmission, with FTIR, a thin film of oils was applied between two KBr plates. The FTIR spectrometer was set to scan in the mid-infrared range (4000 to 400 cm^−1^), which is optimal for detecting organic functional groups, with a resolution of around 4 cm^−1^ to obtain well-defined peaks. Initially, a background scan was performed to eliminate any ambient contributions, which were later subtracted from the sample scan [35]. The in-built software analyzer was used to generate the data sheets.

(f) Sulfur Analysis of Soils

Soil samples from Norman E. Borlaug Crop Research Centre, where the three mustard plants were grown, were checked for their sulfur content [36].

#### 2.2.2. Functional Properties

For NSI, PDI, oils’ FAC, EA and ES [37,38], and whippability and foam stability [39,40], estimation was carried out to elucidate food functional properties.

Every phytochemical and functional property test was carried out in 3 replications and results were explicated as mean ± standard deviation. The statistical analysis of experimental analysis was carried out using SPSS software (V.28, 2021) using ANOVA tool at *p* < 0.05.

#### 2.2.3. Targeted Molecular Docking

##### Recognition of Target Protein and Molecular Docking

**Figure 1:**
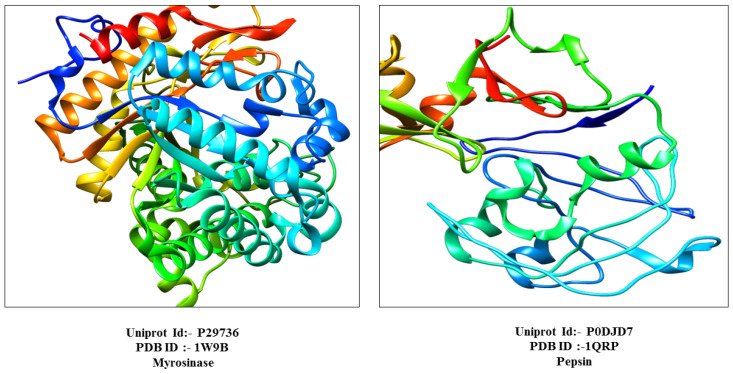
Three-dimensional structure of myrosinase and pepsin with PDB and UniprotID.

The protein myrosinase, and pepsin were identified using their UniProtIDs P29736 and P0DJD7, respectively, via the UniProtKB database. Their crystal structures corresponding to PDB IDs 1W9B for myrosinase and 1QRP for pepsin, as shown in Figure 1, were obtained from the Protein Data Bank (PDB) for structural analysis. These protein structures were selected based on the factors like resolution, R-value, and structural coverage. Their physicochemical properties were then analyzed using the ProtParam tool (https://web.expasy.org/protparam/ (accessed on 12 March 2022)) [41].

**Figure 2:**
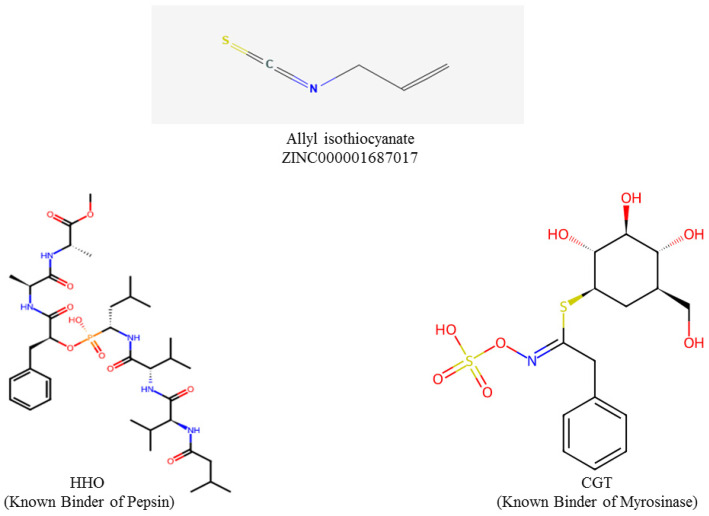
Chemical structure of AITC and known binders (HH0 and CGT).

AITC with ZINC000001687017 was sourced from the ZINC15 database (https://zinc15.docking.org/ (accessed on 16 March 2022)) in SDF format. The Structured Document Format (SDF) files were then converted to Protein Data Bank (PDB) format using the software named Open Babel 2.4.1 [42]. The known binders, Carba-Glucotropaeolin (CGT) for myrosinase (https://www.rcsb.org/ligand/CGT (accessed on 20 March 2022)) and (methylN-[(2S)-2-({(S)-hydroxy[(1R)-3-methyl-1-{[N-(3-methylbutanoyl)-L-valyl-L-valyl]amino}butyl]phosphoryl}oxy)-3-phenylpropanoyl]-L-alanyl-L-alaninate), code name HH0, for pepsin (https://www.rcsb.org/ligand/HH0 (accessed on 22 March 2022)), were retrieved from the Research Collaboratory for Structural Bioinformatics (RCSB), as shown in Figure 2. Molecular docking of AITC was performed to predict its interactions with myrosinase, and pepsin. The protein preparation of both myrosinase and pepsin were carried out with the help of UCSF Chimera, in which non-essential elements such as heteroatoms and water molecules were removed to improve docking accuracy [43,44]. The prepared protein and ligand structures were loaded into PyRx for docking simulations. Subsequently, ligands were converted to PDBQT format and subjected to energy minimization to ensure the enhanced geometry of the molecule and ligand–receptor affinity. Later, a docking grid was defined around the active sites of the proteins, and molecular docking simulations were performed using PyRx [45]. Conformers of AITC were evaluated based on their binding affinities and root mean square deviation (RMSD) values (≤2 Å) to ensure accurate binding predictions.

##### Protein–Protein Molecular Docking

The software High-Accuracy Docking (HDOCK, v2.5-2024.03) was used to conduct blind molecular docking to identify potential binding sites among pepsin, and the napin and cruciferin proteins, aiming to determine the most favorable protein–protein complex configuration. Pepsin, myrosinase, and cruciferin proteins were identified using UniProtKB with the unique UniProtIDs P0DJD7, P29736, and Q7XB53, respectively. These IDs helped confirm the identity of each protein and ensure that the correct structures were used in the study. The crystal structures of respective proteins corresponding to PDB IDs 1QRP (pepsin), 1W9B (myrosinase), and 3KGL (cruciferin) were obtained from the PDB for structural analysis. Pepsin and cruciferin were designated as ligand, and docked with myrosinase, the receptor protein. HDOCK was then utilized to predict the optimal docking pose for the modeled complex based on a structural template.

## 3. Results

Mustard is a plant of culinary importance throughout the world. The mixture of nutritional and anti-nutritional factors makes its use less or more evitable. The edible parts of mustard include its seed and the oils extracted from them. The seed meal left after oil extraction is deprived of major anti-nutritional factors but the leftover presence of glucosinolate breakdown products limits its use as food and, to some extent, as feed for animals.

### 3.1. GBP Estimation and Relation with Pepsin Digestibility in Mustard Seed Meals

The three mustard seed types analyzed here reflect the presence of GBP (Table 1) which affects the protein bioavailability checked through a pepsin digestibility analysis in precipitated protein isolates (Table 2).

Table 1 shows the percent quantity of GBP retained in mustard meals before and after the lowering of the GBPs. Each value is the mean of three observations with *p* ≤ 0.05.

Table 2 reflects the improvement in the pepsin digestibility of proteins after the removal of GBPs from three different mustard seed meals in comparison to the pepsin digestibility of proteins before the lowering of the GBPs, and also depicts the crude protein, moisture, and sinapine content, all expressed in % in three mustard seed meals. Each value is the mean of three observations with *p* ≤ 0.05.

### 3.2. Functional Properties Estimation

The proteinaceous molecules’ behavior in relation to the surface properties, and protein–water and protein–protein interactions has been analyzed here in terms of EA, ES, FAC, FS, whippability, PDI, and ESI (Table 3).

Table 3 governs the functional properties of three different seed meals and draws a comparison among them. Each value is the mean of three observations with *p* ≤ 0.05.

**Figure 3:**
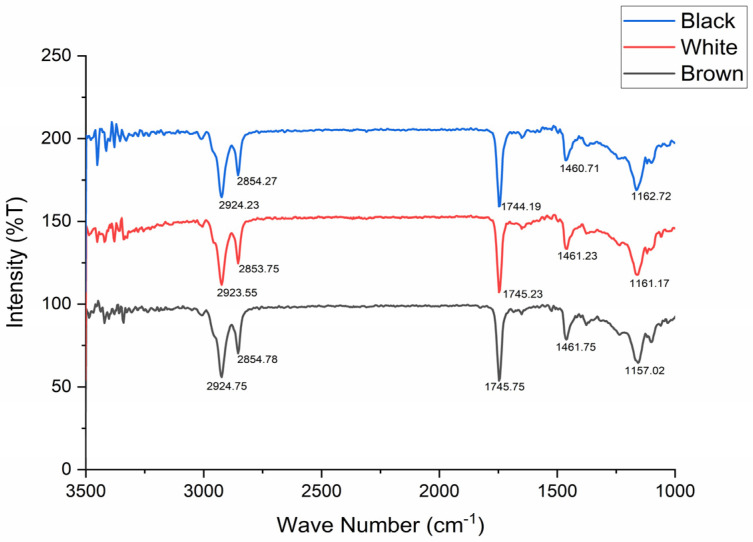
FTIR analysis of mustard oil types. The percent transmittance with a wavenumber (cm^−1^) range of 1000–3500.

The oils extracted from mustard seeds were subjected to an FTIR analysis (Figure 3), revealing the percent transmittance with a wave number (cm^−1^) range of 1000–3500. The presence of carbon–hydrogen single bond asymmetric and symmetric vibrations were noted at 2924.23 cm^−1^ and 2854.27 cm^−1^ for black mustard, 2923.55 cm^−1^ and 2853.75 cm^−1^ for white mustard, and 2924.75 cm^−1^ and 2854.78 cm^−1^ for brown mustard oils. The carbon–oxygen double bond ester carbonyl of triglycerides was noted at 1744.19 cm^−1^ (black mustard), 1745.23 cm^−1^ (white mustard), and at 1745.75 cm^−1^ (brown mustard). The carbon–oxygen single bond was noted with the highest deflections from similar values, as were observed for the previous two bonds for three different mustard oil types. For black mustard (1162.72 cm^−1^), white mustard (1161.17 cm^−1^), and brown mustard, the carbon–oxygen bond peak was observed at 1157.02 cm^−1^.

The protein digestibility in mustard seed meal, before and after the removal of GBPs, improved in all mustard seed types, which increased from 70.84% to 83.68% in brown mustard, and from 63.19% to 75.79% in black mustard, and only a slight increase was noticed in white mustard seed meal from 69.81% to 74.34%. The mustard types were grown under field conditions to harvest seeds, with a narrow range (45.62 to 53.12 mg sulfur/kg soils) of sulfur content variation in soils, so as to check an environmental up-rise in the total glucosinolate content of mustard plants grown (Table 4).

Table 4 demonstrates the presence of sulfur content in soils as mg/Kg (milligram/Kilogram) before the sowing of three mustard types. Each value is the mean of three observations with *p* ≤ 0.05.

### 3.3. Molecular Docking

**Figure 4:**
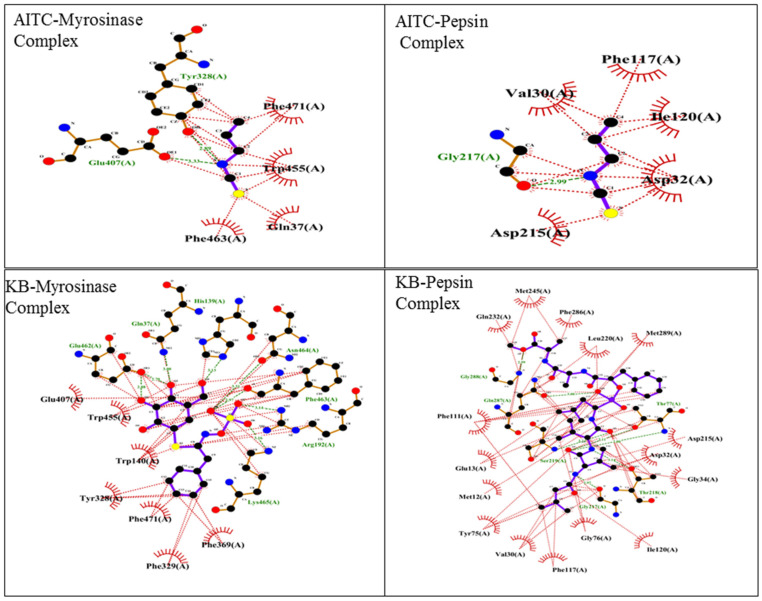
Two-dimensional representations of protein–ligand interactions via Ligplot (+v.2.2).

Molecular docking is an important tool for predicting the orientation and binding affinity of ligands within a protein’s active site. These in silico approaches provide insights into how small molecules interact with biological systems, playing a crucial role in the drug discovery process. Molecular docking was conducted using PyRx to examine the interactions of AITC and two known binders, CGT and HH0, with the proteins pepsin and myrosinase, respectively. The binding free energy results, summarized in Table 5, reveal the strength of these interactions. AITC showed moderate binding affinities, with binding free energies of −3.6 kcal/mol for pepsin and −3.7 kcal/mol for myrosinase. In comparison, the known binders demonstrated stronger interactions, with binding free energies of −8.4 kcal/mol for pepsin and −8.9 kcal/mol for myrosinase, indicating more stable complexes (Table 5). To gain further insight into these interactions, LigPlot was used to create 2D representations of the protein–ligand interactions. These visualizations highlight key intermolecular forces—such as hydrophobic interactions, hydrogen bonding, and electrostatic interactions—that contribute to binding. The interactions are shown in Table 5 and Figure 4, with key residues involved in binding. For instance, AITC’s interaction with pepsin included residues Val130, Phe117, Ile120, Asp32, and Gly217, while its interaction with myrosinase involved residues Phe471, Trp455, and Glu407. Similarly, the known binders interacted with residues Gln232, Met245, and Asp215 in pepsin, and with residues Trp455, Glu407, and Phe471 in myrosinase. This detailed mapping highlights the specific amino acids that stabilize each binding complex (Table 6).

Protein–protein docking was also performed using HDOCK to explore the interactions between pepsin and myrosinase. The results showed a docking score of −229.35, which is relatively low and reflects favorable interactions. The confidence score of 0.8302 indicates high reliability for the predicted binding between pepsin and myrosinase. However, the ligand RMSD value was 78.51 Å, which is unusually high. RMSD values below 2 Å are generally considered well-aligned, so this large deviation suggests potential conformational changes, structural flexibility, or limitations in accurately predicting the binding mode. The LG score of 5.378 further supports the reliability of this model, as scores above 4.0 typically indicate reliable predictions. However, the MaxSub value of 0.197 implies only a moderate structural alignment between the predicted docked structure and the reference, which could reflect flexibility or structural shifts in the interaction. Taken together, these findings indicate a strong binding affinity between pepsin and myrosinase, although further refinement may improve the model accuracy.

**Figure 5:**
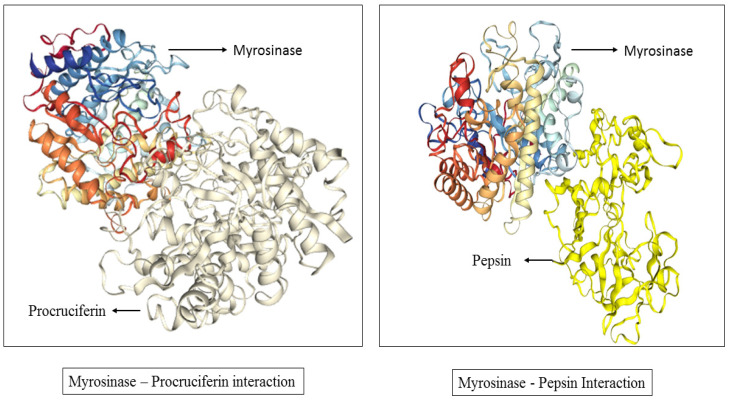
Protein–protein molecular docking via HDOCK.

Similarly, the docking of cruciferin with myrosinase yielded a more favorable docking score of −354.06, indicating an even stronger binding affinity. The confidence score of 0.9834 suggests a very high reliability for the predicted binding mode. Additionally, the ligand RMSD of 0.32 Å indicates a close alignment between the docked and reference structures, signifying a highly accurate prediction of the binding mode. The LG score of 6.284 and the MaxSub value of 0.277 further confirm the reliability and strong structural alignment of this model. Overall, these docking simulations, shown in Table 7 and Figure 5, demonstrate strong binding interactions between myrosinase and both pepsin and cruciferin, with cruciferin showing the strongest predicted binding affinity.

### 3.4. Statistical Analysis

**Figure 6:**
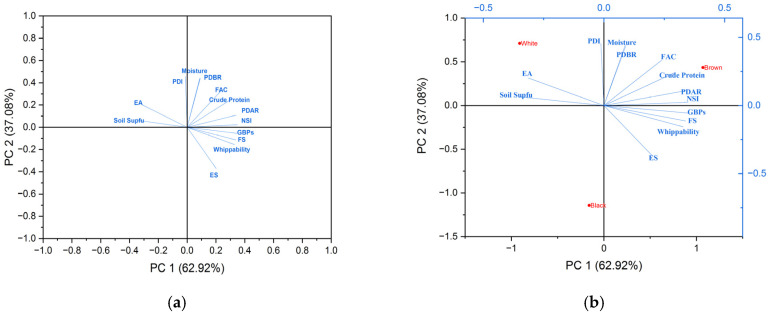
(**a**,**b**) represents loading plot and bi-plot, respectively. The respective eigenvalues and percentage variance were obtained from principal component analysis.

Principal component analysis was utilized for statistical analysis by which the loading plot (Figure 6a) and bi-plot (Figure 6b) were obtained, by using Origin Pro tool. The eigenvalues of the correlation matrix are 8.17 and 4.82 with the percentage of variance as 62.92% and 37.08%, respectively. ANOVA (one-way for Table 1 and Table 4; two-way for Table 2 and Table 3) was applied to find differences between the means of values mentioned in the tables with *p* ≤ 0.05.

## 4. Discussion

The defatted mustard cakes are a likely source of protein for animal kingdom organisms. The current study is the first of its kind with respect to the comparison drawn in biochemical and food functional attributes of three different seed-coat-color mustards, namely, black, brown, and white. The drying of seeds prior to oil extraction made the moisture content lower, with values of 4.12 ± 0.19% (brown), 3.88 ± 0.11 (black), and 4.09 ± 0.08 (white) in comparison to an average of 8.2 ± 0.2% as per previous reports [31], but, here, for undermining phytochemicals like sinapine, moisture needs to be lowered down to 5%. The variation in sinapine content was almost similar in seeds, at 2.19 ± 0.09% (brown), 3.45 ± 0.11% (black), and 2.23 ± 0.14% (white), as was previously reported [3]. Moreover, the pattern of variation was similar, with the highest sinapine content found to be present in black mustard, followed by white mustard, with the lowest in brown mustard. Prior to the pepsin digestibility analysis, the crude protein estimation was carried out with values obtained as follows: 34.29 ± 0.03 (brown), 31.89 ± 0.15 (black), and 32.34 ± 0.04 (white). These values were found to be altogether lower than reported previously [32,46], while some reports suggested an almost equal amount of crude protein concentrate in mustard cakes [47,48,49]. The differences observed may have arisen due to the variety utilized, and it also depends on the harvesting season. The removal of GBP improved overall protein digestibility by pepsin in all three mustard oils cakes. The enzymatic digestibility of the protein improved from 70.84 ± 0.13 to 83.68 ± 0.72 in brown, 63.19 ± 0.24 to 75.79 ± 0.54 in black, and from 69.81 ± 0.17 to 74.34 ± 0.86 in white mustard seed meal, as is reflected in Table 2. This is in accordance with the results obtained by various research groups. The presence of GBP as AITC was also found to coincide [31,49,50]. In accordance to Table 3, different food functional properties were determined which varied slightly as per the previous literature report [51]. The FAC was found to be the highest in brown (167 ± 1.12%), EA the highest in white (63.11 ± 0.23%), ES the highest in black (89.86 ± 1.11%), FS the highest in brown (91± 0.04 mL), and whippability the highest in brown mustard (141.98 ± 1.15%). NSI was found to be the highest in brown mustard seed meal (2.49 ± 0.02), while PDI was found to be the highest for white mustard seed meal (7.12 ± 0.06). The oil content was found to be the highest in white mustard seed (40.23 ± 1.12%), followed by black (36.89 ± 0.87%), with the lowest in brown mustard seed (37.90 ± 1.01), as suggested in previous reports [27,52]. The FTIR analysis of mustard oils extracted has shown a similar range of bond values in accordance with previous findings [53,54]. The sulfur content was found to be similar in the crop research center of Pantnagar as per a previous report [55]. The FTIR analysis reveals a similar kind of bond in the oils of all three mustard types, showing similar functional groups present in these three mustard oils, reflecting an almost uniform fatty acid composition.

As indicated from the results, the AITC binds weakly to pepsin and also to myrosinase. Since pepsin is involved in protein digestion but, as evident from the results, the AITC has a minimalistic role in hampering the activity of pepsin, thereby, it competes with the protein cruciferin in binding on the active site of pepsin, suggesting a possible slowing of protein digestion in vivo. That is why its consumption carried out in minimal concentrations is not harmful, and it is also known that AITC is an anti-cancerous, anti-oxidizing, and anti-inflammatory agent promoting gut health, and, thus, cannot be ignored in diet through the intake of mustard seeds and seed meals [56]. Moreover, AITC binds weakly to myrosinase, suggesting that AITC itself, being a metabolic product of a myrosinase-catalyzed reaction, may competitively inhibit it at higher concentrations over time and may inhibit its function, as it is released when sinigrin is catalyzed by myrosinase. The regulation of myrosinase is evident from previous reports as sulfate (also a product of a myrosinase-catalyzed reaction) acts as a competitive inhibitor of myrosinase isolated from *Raphanus sativus* seedlings, which was shown to have an inhibitory constant of 60 and 27 mM with respect to sinigrin (the source metabolite of AITC) and ascorbate, respectively [57,58]. Thus, future insights are required for determining the AITC-involved metabolic pathways of glucosinolate–myrosinase under an in vivo system. The docking score of pepsin–myrosinase was found to be −229.35 and that of cruciferin–myrosinase was −354.06, suggesting that the cruciferin–myrosinase complex results in a more stable and favorable interaction compared to the pepsin–myrosinase complex. This implies that cruciferin has a more significant impact on myrosinase regulation than pepsin. Future insights are required on how the glucosinolate-catalyzing enzyme, myrosinase, interferes with the bio-availability of cruciferin, the major protein of mustard seed and seed meals. The RMSD of <2 Å suggests a good docking score [59], and, here, the RMSD of cruciferin–myrosinase came out to be 0.32 °A, while pepsin–myrosinase represented a value of 78.51 Å, suggesting cruciferin serves as a substrate of enzyme myrosinase, while pepsin, being an enzyme itself, cannot be a substrate for another enzyme, myrosinase. However, the confidence score was noted as 0.8302 and 0.9834 for pepsin–myrosinase and cruciferin–myrosinase, respectively, suggesting these molecules would very likely bind, as a >0.7 confidence score represents likely binding [60,61,62,63,64,65,66]. Thus, more deep scientific findings are required to be discovered in the future to reveal the binding interaction between pepsin–myrosinase.

## 5. Conclusions

The present study described the influence of anti-nutritional factors, especially glucosinolate breakdown products (measured titrimetrically), with respect to changes in the enzymatic digestion of protein by pepsin and the food functionality of mustard estimated in precipitated protein isolates recovered from mustard seed meals. The FTIR analysis of three mustard oils revealed the presence of a similar kind of chemical bond showing the similar and comparable fatty acid composition, which is a cause of their viscous nature. This research highlights the fundamental aspects towards food with a comparison drawn among black, brown, and white mustard seed types. Brown-mustard-seed-meal-derived PPI showed the highest values for FAC, whippability, FS, and NSI. In contrast, EA and PDI were the highest in white mustard, while black mustard exhibited the highest ES value. This reflects that, although the functional properties are found to be highest in brown mustard seed, at the same time, brown mustard seed has highest total glucosinolate content compared to the other mustard seed meals analyzed here, limiting its use as a high-quantity food product. On the other hand, white mustard seed possesses the lowest total glucosinolate content but also comes with the lowest values of functional properties that make its utilization as food inappropriate in larger consumption amounts. Interestingly, black mustard seed possesses middle-scale values of total glucosinolates and functional properties among brown and white mustard, but the highest sinapine content is found in black mustard seed compared to white and brown mustard, which limits its use as food in larger amounts. The improvement in overall protein digestibility was noticed to be the highest in brown and black mustard seed meal at similar scales, and the lowest improvement was noticed in white mustard seed meal. The rationale behind this study is to correlate the biochemical and functional parameters among brown, black, and white mustard’s seed and oils, the consumption of which is limited by the presence of anti-nutritional factors. Moreover, docking interactions predict the binding interactions among pepsin, myrosinase, and AITC. This reveals the fact that glucosinolates interfere in protein’s bio-availability which interrupts the higher consumption of mustard seeds in diet in large proportions.

In this investigation, tools like LigPlot offered a further visualization of molecular interactions, pinpointing specific residues involved in hydrogen bonding and hydrophobic contacts, which are crucial for understanding binding dynamics. Additionally, HDOCK’s docking score between pepsin and myrosinase suggested robust protein–protein interaction, albeit with high RMSD values, potentially indicating conformational variability or flexibility within the ligand-binding poses. Lower RMSD values, such as those in cruciferin’s docking with myrosinase, supported a reliable and stable complex formation.

To evaluate the low consumption of globally utilized mustard seeds besides their oils, the leftover seed meals of three different mustard types are being analyzed here with the variation observed among their food functionality and the biochemical factors involved. All these mustard seed meals are limited in their consumption besides their higher protein content. The responsible factors governing their limited use as food are altogether different in their content as well as in their class. Black mustard seed meal usage is limited by the higher presence of sinapine esters, while brown mustard possesses the highest level of glucosinolates, and white mustard has the lowest-scale values of food functional attributes. The glucosinolates and GBP limit protein’s bio-availability as is reflected by the protein digestibility analysis by pepsin, and the food functional attributes change with the specific mustard type. In future, there is a need to enhance the metabolic efficiency of mustard by lowering the anti-nutritional factors and, thereby, studying food functional attributes on the biochemical pathway scale in order to make the utilization of mustard seed meal as a food source possible, which, otherwise, is not used as potential food in its present form. These findings are crucial for understanding the interactions among the protein and metabolites, particularly in the context of their biological functions, and provides potential implications for the enzyme activity in metabolic pathways involving glucosinolates and their derivatives. The docking scores may also guide future studies aimed at characterizing the mechanistic details of these interactions and their functional consequences in vivo. They could also inform future research aimed at elucidating the functional roles of cruciferin, pepsin, myrosinase, and AITC in plant metabolism and their potential applications in agricultural and pharmaceutical contexts. 

## Figures and Tables

**Table 1:** Determination of GBP in mustard cakes before and after their removal.

Determination of GBP in Mustard Cakes Before and After Their Removal		
Mustard Meal	% GBP Before Their Removal	% GBP After Their Removal
Brown Mustard	0.91 ± 0.005	0.32 ± 0.003
Black Mustard	0.74 ± 0.006	0.31 ± 0..002
White Mustard	0.58 ± 0.008	0.30 ± 0.004

**Table 2:** Protein digestibility analysis in GBP lowered and non-lowered mustard seed meals with estimation of crude protein, moisture, and sinapine content.

Parameters Analyzed	Brown Mustard Meal	Black Mustard Meal	White Mustard Meal
Crude protein (%)	34.29 ± 0.03	31.89 ± 0.15	32.34 ± 0.04
Moisture content (%)	4.12 ± 0.19	3.88 ± 0.11	4.09 ± 0.08
Sinapine content (%)	2.19 ± 0.09	3.45 ± 0.11	2.23 ± 0.14
Pepsin digestibility (%) analysis before lowering of GBPs	70.84 ± 0.13	63.19 ± 0.24	69.81 ± 0.17
Pepsin digestibility (%) analysis after lowering of GBPs	83.68 ± 0.72	75.79 ± 0.54	74.34 ± 0.86

**Table 3:** Functional properties of mustard seed meals.

Functional Properties of Mustard Seed Meals								
Meal Type	Fat Absorption Capacity (%)	Emulsifying Activity (%)	Emulsion Stability (%)	Whippability (%)	Foam Stability (mL) @ 140Min	NSI	PDI	Oils %
Brown Mustard Meal	167 ± 1.12	51.96 ± 0.76	86.72 ± 1.21	141.98 ± 1.15	91 ± 0.04	2.49 ± 0.02	6.93 ± 0.02	37.90 ± 1.01
Black Mustard Meal	148 ± 1.05	54.32 ± 0.56	89.86 ± 1.11	132.67 ± 1.26	89 ± 0.06	2.23 ± 0.04	6.09 ± 0.05	36.89 ± 0.87
White Mustard Meal	156 ± 1.34	63.11 ± 0.23	79.24 ± 1.03	112.32 ± 1.22	86 ± 0.08	2.10 ± 0.02	7.12 ± 0.06	40.23 ± 1.12

**Table 4:** Sulfur content of soils under mustard cultivation.

Mustard Type	Soil Sulfur Content (mg/Kg)
Brown Mustard	53.12 ± 0.05
Black Mustard	50.37 ± 0.03
White Mustard	45.62 ± 0.08

**Table 5:** Binding free energy (kcal/mol) of AITC and known binders with GST and ST.

Name of the Ligand	Binding Free Energy (kcal/mol)
AITC–Pepsin	−3.6
AITC–Myrosinase	−3.7
KB(Pepsin)	−8.4
KB(Myrosinase)	−8.9

**Table 6:** Representation of the residue of GST and ST that interacts with selected ligands.

Ligands	Residues
AITC–Pepsin	Val130, Phe117, Ile120, Asp32, Asp215, GLy217, Asp32, GLy34, Thr218, Gly217, Ile120, Gly76, Phe117, Val130, Tyr75, Met12, Glu13, Phe111, Gln287, Gly288
AITC–Myrosinase	Phe471, Trp455, Gln37, Phe463, Glu407, Tyr328
KB(Pepsin)	Gln232, Met245, Phe286, Leu220, Met289, Asp215, Thr77
KB(Myrosinase)	Glu407, Trp455, Trp140, Trp328, Phe471, Phe369, Lys465, Arg192, Phe463, Asn464, His139, Gln37, Glu462

**Table 7:** Protein–protein molecular docking scores via HDOCK.

Rank	Docking Score	Confidence Score	Ligand rmsd (Å)	LGscore	MaxSub
Pepsin–Myrosinase	−229.35	0.8302	78.51	5.378	0.197
Cruciferin–Myrosinase	−354.06	0.9834	0.32	6.284	0.277

## Data Availability

The data presented in this study are available on request from the corresponding authors. The data are not publicly available due to privacy restrictions.

## References

[1] Golubkina N., Kekina H., Caruso G. (2018). Yield, Quality and Antioxidant Properties of Indian Mustard (*Brassica juncea* L.) in Response to Foliar Biofortification with Selenium and Iodine. Plants.

[2] Givnish T.J. (2023). Plant biology: Phylogenomics of mustards and their relatives. Curr. Biol..

[3] Garg S., Pant U., Nain P., Punetha H. (2023). Nutritional and Anti-Nutritional and Anti-Oxidative Profiling of Globally Utilized Diverse Seed Coat Color Mustards. Biol. Forum.

[4] Lietzow J. (2021). Biologically Active Compounds in Mustard Seeds: A Toxicological Perspective. Foods.

[5] Sarker A.K., Saha D., Begum H., Zaman A., Rahman M.M. (2015). Comparison of Cake Compositions, Pepsin Digestibility, and Amino Acids Concentration of Proteins Isolated from Black Mustard and Yellow Mustard Cakes. AMB Express.

[6] Barba F.J., Nikmaram N., Roohinejad S., Khelfa A., Zhu Z., Koubaa M. (2016). Bioavailability of Glucosinolates and Their Breakdown Products: Impact of Processing. Front. Nutr..

[7] Pałgan K., Żbikowska-Gotz M., Bartuzi Z. (2018). Dangerous anaphylactic reaction to mustard. Arch. Med. Sci..

[8] Falk K.L., Tokuhisa J.G., Gershenzon J. (2007). The Effect of Sulfur Nutrition on Plant Glucosinolate Content: Physiology and Molecular Mechanisms. Plant Biol..

[9] He S. (2023). Study on Physicochemical Properties of Food Protein. Molecules.

[10] Perera S.P., McIntosh T.C., Wanasundara J.P. (2016). Structural Properties of Cruciferin and Napin of *Brassica napus* (Canola) Show Distinct Responses to Changes in pH and Temperature. Plants.

[11] Höglund A.S., Rödin J., Larsson E., Rask L. (1992). Distribution of napin and cruciferin in developing rape seed embryos. Plant Physiol..

[12] Temple N.J. (2022). A rational definition for functional foods: A perspective. Front. Nutr..

[13] Galanakis C.M. (2021). Functionality of Food Components and Emerging Technologies. Foods.

[14] Chandra S., Singh S., Kumari D. (2015). Evaluation of Functional Properties of Composite Flours and Sensorial Attributes of Composite Flour Biscuits. J. Food Sci. Technol..

[15] Costa C., Medronho B., Filipe A., Mira I., Lindman B., Edlund H., Norgren M. (2019). Emulsion Formation and Stabilization by Biomolecules: The Leading Role of Cellulose. Polymers.

[16] Einhorn-Stoll U., Weiss M., Kunzek H. (2002). Influence of the Emulsion Components and Preparation Method on the Laboratory-Scale Preparation of o/w Emulsions Containing Different Types of Dispersed Phases and/or Emulsifiers. Food/Nahrung.

[17] Camacho-Chab J.C., Guézennec J., Chan-Bacab M.J., Ríos-Leal E., Sinquin C., Muñiz-Salazar R., Rosa-García D., Reyes-Estebanez M., Ortega-Morales B.O. (2013). Emulsifying Activity and Stability of a Non-Toxic Bioemulsifier Synthesized by Microbacterium sp. MC3B-10. Int. J. Mol. Sci..

[18] Ricardo F., Pradilla D., Cruz J.C., Alvarez O. (2021). Emerging Emulsifiers: Conceptual Basis for the Identification and Rational Design of Peptides with Surface Activity. Int. J. Mol. Sci..

[19] Freitas F., Alves V., Carvalheira M., Costa N., Oliveira R., Reis M. (2009). Emulsifying behaviour and rheological properties of the extracellular polysaccharide produced by *Pseudomonas oleovorans* grown on glycerol byproduct. Carbohydr. Polym..

[20] Ma S., Wang H., Dou Y., Liang X., Zheng Y., Wu X., Xue M. (2022). Anti-Nutritional Factors and Protein Dispersibility Index as Principal Quality Indicators for Soybean Meal in Diet of Nile Tilapia (*Oreochromis niloticus* GIFT), a Meta-Analysis. Animals.

[21] Khalil M., Ragab F.R. (1985). Some Functional Properties of Oilseed Proteins. Food/Nahrung.

[22] Zhu J., Lu F., Liu D., Zhao X., Chao J., Wang Y., Luan Y., Ma H. (2024). The process of solid-state fermentation of soybean meal: Antimicrobial activity, fermentation heat generation and nitrogen solubility index. J. Sci. Food Agric..

[23] Batal A.B., Douglas M.W., Engram A.E., Parsons C.M. (2000). Protein Dispersibility Index as an Indicator of Adequately Processed Soybean Meal. Poult. Sci..

[24] Sensoy I. (2021). A Review on the Food Digestion in the Digestive Tract and the Used In Vitro Models. Curr. Res. Food Sci..

[25] Campos L.A., Sancho J. (2003). The Active Site of Pepsin is formed in the Intermediate Conformation Dominant at Mildly Acidic pH. FEBS Lett..

[26] Stojanović Z.S., Uletilović D.D., Kravić S.Z., Kevrešan Z.S., Grahovac N.L., Lončarević L.S., Đurović A.D., Marjanović-Jeromela A.M. (2023). Comparative Study of the Nutritional and Chemical Composition of New Oils Rape, Safflower and Mustard Seed Varieties Developed and Grown in Serbia. Plants.

[27] Ye Q., Meng X. (2022). Highly efficient authentication of edible oils by ftir spectroscopy coupled with chemometrics. Food Chem..

[28] Wang W., Wang X., Ye H., Hu B., Zhou L., Jabbar S., Shen W. (2016). Optimization of extraction, characterization and antioxidant activity of polysaccharides from *Brassica rapa* L.. Int. J. Biol. Macromol..

[29] Kolodziejczyk P.P., Wang X., Marianchuk M., Lu W., Amarowicz R. (1999). Phenolics in rapeseed: Capillary electrophoresis as a novel analytical method for detection of sinapine, sinapic acid esters and ferulates. Quantum.

[30] Singh U. (1988). Antinutritional Factors of Chickpea and Pigeonpea and Their Removal by Processing. Plant Foods Hum. Nutr..

[31] Marnoch R., Diosady L.L. (2006). Production of Mustard Protein Isolates from Oriental Mustard Seed (*Brassica juncea* L.). J. Am. Oils Chem. Soc..

[32] Dhaliwal S.S., Sharma V., Shukla A.K., Kaur M., Verma V., Sandhu P.S., Alsuhaibani A.M., Gaber A., Hossain A. (2022). Biofortification of Oils Quality, Yield, and Nutrient Uptake in Indian Mustard (*Brassica juncea* L.) by Foliar Application of Boron and Nitrogen. Front. Plant Sci..

[33] Zhang K., Wen Q., Wang Y., Li T., Nie B., Zhang Y. (2022). Study on the In Vitro Digestion Process of Green Wheat Protein: Structure Characterization and Product Analysis. Food Sci. Nutr..

[34] Mertz E.T., Hassen M.M., Cairns-Whittern C., Kirleis A.W., Tu L., Axtell L. (1984). Pepsin Digestibility of Proteins in Sorghum and Other Major Cereals. Proc. Natl. Acad. Sci. USA.

[35] Jamwal R., Amit, Kumari S., Balan B., Kelly S., Cannavan A., Singh D.K. (2021). Rapid and non-destructive approach for the detection of fried mustard oils adulteration in pure mustard oils via ATR-FTIR spectroscopy-chemometrics. Spectrochim. Acta Part A Mol. Biomol. Spectrosc..

[36] Shukla A.K., Behera S.K., Singh V.K., Prakash C., Sachan A.K., Dhaliwal S.S., Srivastava P.C., Pachauri S.P., Tripathi A., Pathak J. (2020). Pre-Monsoon Spatial Distribution of Available Micronutrients and Sulphur in Surface Soils and Their Management Zones in Indian Indo-Gangetic Plain. PLoS ONE.

[37] Séré A., Bougma A., Bazié B.S.R., Traoré E., Parkouda C., Gnankiné O., Bassolé I.H.N. (2021). Chemical Composition, Energy and Nutritional Values, Digestibility, and Functional Properties of Defatted Flour, Protein Concentrates, and Isolates from *Carbula marginella* (Hemiptera: Pentatomidae) and *Cirina butyrospermi* (Lepidoptera: Saturniidae). BMC Chem..

[38] Tan S.H., Mailer R.J., Blanchard C.L., Agboola S.O. (2011). Canola Proteins for Human Consumption: Extraction, Profile, and Functional Properties. J. Food Sci..

[39] Yan G., Wang S., Li Y., Zhang J., Ding H., Li Y., Zhang L. (2022). Effect of Different Polymerization Degrees and Fatty Acids of Polyglycerol Esters on the Physical Properties and Whippability of Recombined Dairy Cream. Foods.

[40] Lin M.J.Y., Humbert E.S., Sosulki F.W. (1974). Certain functional properties of sunflower meal products. J. Food Sci..

[41] Garg V.K., Avashthi H., Tiwari A., Jain P.A., Ramkete P.W., Kayastha A.M., Singh V.K. (2016). MFPPI—Multi FASTA ProtParam Interface. Bioinformation.

[42] Yoshikawa N., Hutchison G.R. (2019). Fast, efficient fragment-based coordinate generation for Open Babel. J. Cheminform..

[43] Bitencourt-Ferreira G., de Azevedo W.F. (2019). How Docking Programs Work. Methods Mol. Biol..

[44] Pettersen E.F., Goddard T.D., Huang C.C., Couch G.S., Greenblatt D.M., Meng E.C., Ferrin T.E. (2004). UCSF Chimera—A visualization system for exploratory research and analysis. J. Comput. Chem..

[45] Dallakyan S., Olson A.J. (2015). Small-molecule library screening by docking with PyRx. Methods Mol. Biol..

[46] Shahbazi F. (2013). Aerodynamic Properties of Wild Mustard (*Sinapis arvensis* L.) Seed for Separation from Canola. J. Sci. Food Agric..

[47] Prapakornwiriya N., Diosady L.L. (2004). Isolation of Yellow Mustard Proteins by a Microfiltration-Based Process. Int. J. Appl. Sci. Eng..

[48] Mahima V., Kumar S.K., Tomar D., Roy M., Kumar M. (2015). Effect of Varying Levels of Formaldehyde Treatment of Mustard Oils Cake on Rumen Fermentation, Digestibility in Wheat Straw Based Total Mixed Diets In Vitro. Vet. World.

[49] Aparicio-Saguilán A., Valera-Zaragoza M., Perucini-Avendaño M., Páramo-Calderón D.E., Aguirre-Cruz A., Ramírez-Hernández A., Bello-Pérez L.A. (2015). Lintnerization of Banana Starch Isolated from Underutilized Variety: Morphological, Thermal, Functional Properties, and Digestibility. CyTA-J. Food.

[50] Sun M., Mu T., Zhang M., Arogundade L.A. (2012). Nutritional Assessment and Effects of Heat Processing on Digestibility of Chinese Sweet Potato Protein. J. Food Compos. Anal..

[51] Aboulfadi M., Bdry-El N., Amaar M. (2011). Nutritional and Chemical Evaluation for Two Different Varieties of Mustard Seeds. World Appl. Sci. J..

[52] Sawicka B., Kotiuk E., Kiełtyka-Dadasiewicz A., Krochmal-Marczak B. (2020). Fatty Acids Composition of Mustard Oils from Two Cultivars and Physico-Chemical Characteristics of the Seeds. J. Oleo Sci..

[53] Zahir E., Mehwish R.S., Hameed A., Yousuf A. (2014). Study of Physicochemical Properties of Edible Oils and Evaluation of Frying Oils Quality by Fourier Transform-Infrared (FT-IR) Spectroscopy. Arab. J. Chem..

[54] Borowicz M., Isbrandt M., Paciorek-Sadowska J., Sander P. (2023). Comparing the Properties of Bio-Polyols Based on White Mustard (*Sinapis alba*) Oils Containing Boron and Sulfur Atoms Obtained by Various Methods and Checking Their Influence on the Flammability of Rigid Polyurethane/Polyisocyanurate Foams. Materials.

[55] Singh S.P., Singh R., Srivastava P.C., Singh P. (2009). Different forms of sulphur in soils of Udham singh nagar district, Uttarakhand and their relationship with soil properties. Agropedology.

[56] Zhang Y. (2010). Allyl isothiocyanate as a cancer chemopreventive phytochemical. Mol. Nutr. Food Res..

[57] Bhat R., Vyas D. (2019). Myrosinase: Insights on Structural, Catalytic, Regulatory, and Environmental Interactions. Crit. Rev. Biotechnol..

[58] Shikita M., Fahey J.W., Golden T.R. (1999). An unusual case of “uncompetitive activation” by ascorbic acid: Purification and kinetic properties of a myrosinase from Raphanus sativus seedlings. Biochem. J..

[59] Torres P.H.M., Sodero A.C.R., Jofily P., Silva F.P. (2019). Key Topics in Molecular Docking for Drug Design. Int. J. Mol. Sci..

[60] Zavodszky M.I., Stumpff-Kane A.W., Lee D.J., Feig M. (2009). Scoring confidence index: Statistical evaluation of ligand binding mode predictions. J. Comput. Aided Mol. Des..

[61] Dong X., Deng P., Wang X., Peng C., Peng L. (2024). Structural characteristics and immunomodulatory effects of polysaccharides extracted from plant seeds: A review. Trends Food Sci. Technol..

[62] Zhang Y., Geng Q., Song M., Li X., Yang A., Tong P., Wu Z., Chen H. (2024). The structure and potential allergenicity of peanut allergen monomers after roasting. Food Funct..

[63] Han Y., Zhang Y., Yang Z., Zhang Q., He X., Song Y., Tian L., Wu H. (2024). Improving aerobic digestion of food waste by adding a personalized microbial inoculum. Curr. Microbiol..

[64] Ju Q., Wu X., Li B., Peng H., Lippke S., Gan Y. (2024). Regulation of craving training to support healthy food choices under stress: A randomized control trial employing the hierarchical drift-diffusion model. Appl. Psychol. Health Well. Being.

[65] Xiong J., Chen F., Zhang J., Ao W., Zhou X., Yang H., Wu Z., Wu L., Wang C., Qiu Y. (2022). Occurrence of aflatoxin M1 in three types of milk from Xinjiang, China, and the risk of exposure for milk consumers in different age-sex groups. Foods.

[66] Xiong J., Wen D., Zhou H., Chen R., Wang H., Wang C., Wu Z., Qiu Y., Wu L. (2022). Occurrence of aflatoxin M1 in yogurt and milk in central-eastern China and the risk of exposure in milk consumers. Food Control.

